# Aortic valve repair for the treatment of rheumatic aortic valve disease: a systematic review and meta-analysis

**DOI:** 10.1038/s41598-021-04040-x

**Published:** 2022-01-13

**Authors:** Meng Zhao, Yihu Tang, Luo Li, Yawei Dai, Jieyu Lu, Xiang Liu, Jingxin Zhou, Yanhu Wu

**Affiliations:** 1 Department of Cardiovascular Surgery, The First Affiliated Hospital of Nanjing Medical University, Nanjing, 210029 Jiangsu Province China; 2 Department of Cardiology, The First Affiliated Hospital of Nanjing Medical University, Nanjing, Jiangsu China

**Keywords:** Cardiac device therapy, Interventional cardiology

## Abstract

Valvuloplasty for rheumatic aortic valve disease remains controversial. We conducted this study to explore whether aortic valvuloplasty is appropriate for the rheumatic population. A comprehensive search was conducted, and 7 eligible retrospective studies were identified from PubMed, Embase, Medline and Cochrane (up to April 7, 2020) according to the inclusion and exclusion criteria. The data for hospital mortality, 5-year survival, 5-year reoperation, aortic insufficiency grade (AIG) and aortic valve gradient (AVG) were extracted by 2 independent reviewers and were analysed to evaluate the safety and availability of aortic valvuloplasty for rheumatic patients. The heterogeneity of the results was estimated using the Q test and I^2^ statistics. The fixed pooling model was used when I^2^ ≤ 50%; otherwise, the random pooling model was selected. 7 articles with 418 patients were included. The pooled hospital mortality, 5-year survival and 5-year reoperation rates were 3.2%, 94.5% and 9.9%, respectively. The heterogeneities of the weighted mean differences (WMD) values of the AIG and AVG between preoperation and postoperation were extremely high (I^2^ = 81.5%, *p* < 0.001 in AIG, I^2^ = 97.6%, *p* = 0.003 in AVG). Subgroup analysis suggested that the AIG and AVG were improved by 3.03 grades (I^2^ = 0%, *p* < 0.001) and 3.16 mmHg (I^2^ = 0%, *p* < 0.001) in the European group, respectively. In the Asian group, the AIG and AVG were improved by 2.57 grades (I^2^ = 0%, *p* < 0.001) and 34.39 mmHg (I^2^ = 0%, *p* < 0.001), respectively. Compared with the values at discharge, the AIG was increased by 0.15 grades (I^2^ = 0%, *p* = 0.031) and the AVG was still decreased by 2.07 mmHg (I^2^ = 0%, *p* = 0.031) at the time of follow up. Valvuloplasty is safe and effective to treat rheumatic aortic insufficiency and stenosis, and the duration of maintenance required to improve stenosis was longer than that of insufficiency.

## Introduction

Presently, rheumatic valve disease remains the main reason for cardiac valve surgery^1^. Valve replacement is the traditional surgery for rheumatic valve disease. However, the problem of lifelong anticoagulation in the mechanical group and expensive cost and limited durable years in bioprosthetic group^2^ have made valve replacement surgery a rejected choice for these patients who prefer to pursue high quality of life, especially young patients. Therefore, valvuloplasty surgery becomes a better choice for these patients.

Rheumatic mitral valve repair has been demonstrated to be feasible and has demonstrated good midterm results^3^. The standardization, reproducibility, stable long-term results of mitral repair^4^ and improvement of ventricular remodelling^5^ have made this technique become accepted globally. However, aortic valve repair remains a challenging technique due to its special characteristics of anatomy and histopathology. The cusp extension developed by Duran et al.^6^ is the most classical surgery for cusp retraction. To solve different malformations of the aortic valve, an increasing number of operative procedures, such as free edge unrolling, subcommissural annuloplasty, commissurotomy and supra-aortic crest enhancement, has been developed. Until now, attempts to improve aortic valvuloplasty techniques persist^7^. Except for the difficulty of surgery, success rate of surgery, reoperation rate and whether aortic valvuloplasty can improve the aortic stenosis and insufficient also made aortic valvuloplasty a controversial technique for treating rheumatic aortic disease.

Several studies have reported on aortic valvuloplasty and the follow-up outcomes of patients with rheumatic disease^4,9–14^. However, the results of those studies have not been systemically reviewed and analysed. Thus, we collected and summarized those studies to explore whether aortic valvuloplasty is appropriate for the rheumatic population.

## Materials and methods

### Literature search

A comprehensive non-MeSH search using [(aortic valve repair) OR (aortic valve reconstruction) OR (aortic valvuloplasty) OR (aortic valve remodelling) OR (aortic valve sparing) OR (aortic valve preservation)] AND [(rheumatic patient) OR (rheumatic population) OR (rheumatism) OR (rheumatic heart disease) OR (rheumatic valvular disease)] was conducted by 2 authors independently (Meng Zhao and Yihu Tang) using PubMed, Embase, Medline and Cochrane (up to April 7, 2020), and the language was limited to English.

### Selection criteria

The studies searched in four databases were included by the following criteria: (1) patients had obvious evidence from cardiac ultrasound of rheumatic valve disease andrheumatic lesion affected aortic valve severely and surgical treatment was needed with or without mitral or tricuspid valve involvement; (2) aortic valvuloplasty conducted with or without procedures on the mitral or tricuspid valve; (3) sufficient data were provided for hospital mortality, 5-year survival, 5-year reoperation and AIG and AVG of aortic valve at preoperation, discharge and follow up. Additionally, unqualified studies were excluded by the following criteria: (1) case reports, reviews, editorials and letters; (2) duplicate records; (3) studies with insufficient hospital mortality, 5-year survival, 5-year reoperation rate.

### Data extraction and quality assessment

Two authors extracted necessary information from each included study: first author, publication year, continent, sample size, mean age ± SD, and time of testing of mean AIG and AVG. The quality of each study was assessed by the Newcastle–Ottawa Quality Assessment Scale (NOS). In NOS, article types (RCT: 3 points, cohort study: 2 points, retrospective study: 1 point), comparison (yes: 1 point, no: 0 point), data integrity (yes: 2 points, no: 1 point), follow-up completed (yes:1 point, no: 0 point) and follow-up time (> 10 years: 3 points, 5 years to 10 years: 2 points, < 5 years: 1 point) are the domain scoring point.

### Statistical analysis

The hospital mortality, 5-year survival and 5-year reoperation of each study were pooled to explore the safety and feasibility of the aortic valvuloplasty in the rheumatic population. The WMD values of AIG and AVG between preoperation and postoperation were calculated to demonstrate the validity and effectiveness of the surgery. However, because different continents could have inconsistent medical levels, calculating the WMD values of AIG and AVG directly would result in great heterogeneity. Thus, we conducted subgroup analysis based on the continents. The WMD values of AIG and AVG between discharge and follow up were calculated by STATA to assess the duration of the operation in improving aortic insufficiency and stenosis. The heterogeneity of the results was estimated using the Q test and I^2^ statistics. The fixed pooling model was used when I^2^ ≤ 50%; otherwise, the random pooling model was selected. WMD < 0 suggested improvement in the aortic situation than before, and WMD > 0 suggested deterioration. Leave-one-out sensitivity analysis was conducted to evaluate the robustness of the results. These calculations were completed using STATA v.16 software.

## Results

### Characteristics of the included studies

**Figure 1:**
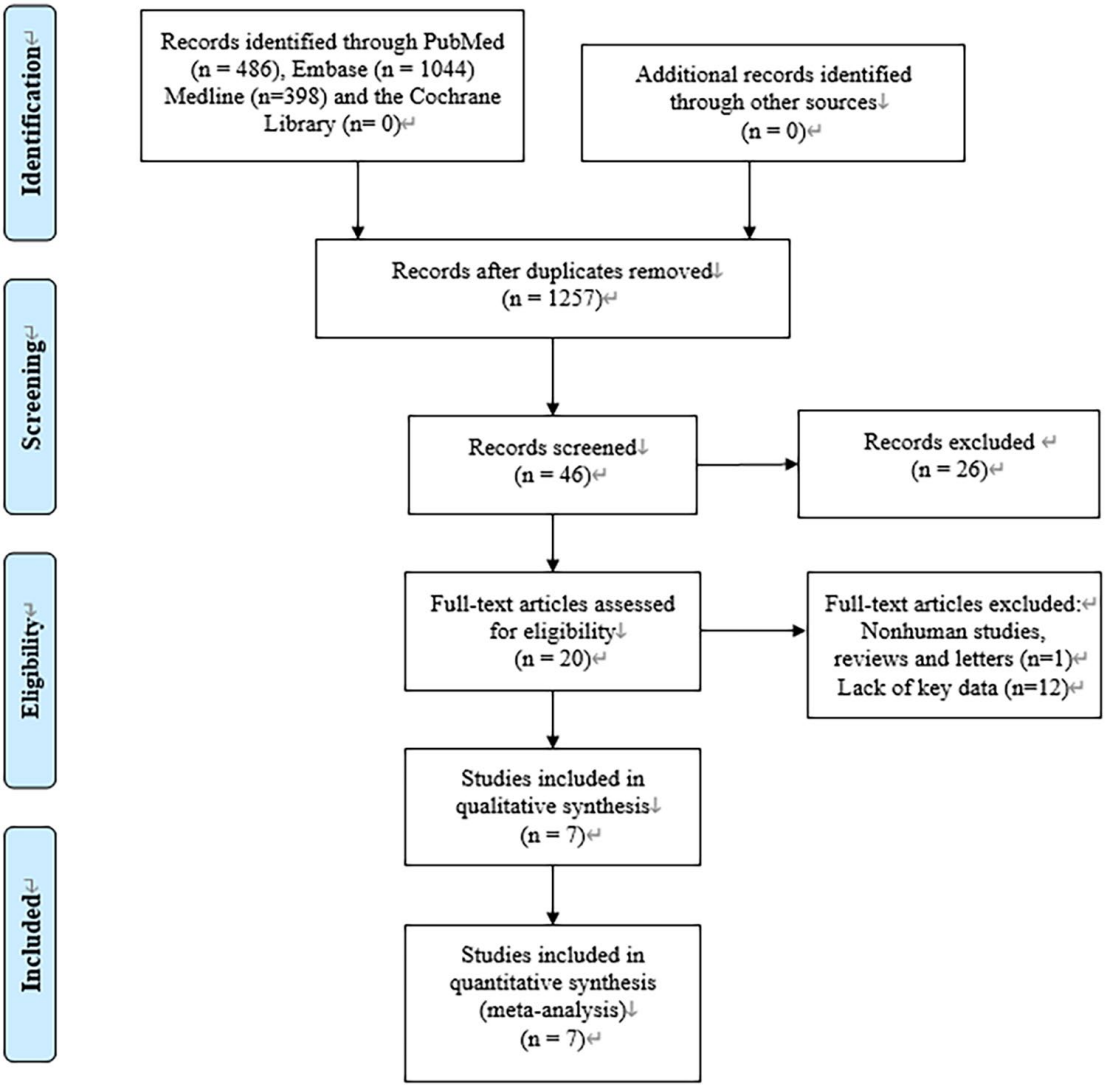
Flow diagram of this systematic review and meta-analysis.

The procedures used to screen eligible studies are shown in Fig. 1^8^. 7 articles with 418 patients published between 1988 and 2010 were included. The first authors’ names were as follows: Jean-Michel Grinda^9^, Afksendiyos Kalangos^4^, Sachin Talwar^10^, C.M. G. Durba^11^, Nilgün Bozbuga^12^, José M. Bernal^13^, and Patrick O. Myers^14^. The data of 5 studies came from Europe and 2 from Asia. Before the operation, all the studies tested the patients for AIG and 3 of them tested for AVG. After the operation, 6 studies tested for AIG and 3 tested for AVG. The details of the 7 included studies are summarized in Table 1.

**Table 1:** Characteristics of the included studies.

First author	Year	Location	Sample size	Mean age ± SD	survival	Time of testing mean AIG	Time of testing mean AVG
Jean-Michel Grinda	2002	Europe	89	16 ± 5	OS	Preoperation, discharge and follow up	NA
Afksendiyos Kalangos	1998	Europe	41	11.5 ± 2.7	OS	Preoperation, discharge and follow up	Preoperation, discharge and follow up
Sachin Talwar	2005	Asia	61	23.7 ± 9.3	OS	Preoperation and follow up	NA
C.M. G. Durba	1988	Europe	50	39.5	OS	Preoperation	NA
Nilgün Bozbuga	2004	Asia	46	31.5 ± 12.2	OS	Preoperation, discharge and follow up	Preoperation, discharge and follow up
José M. Bernal	1997	Europe	53	40.8 ± 11.6	OS	Preoperation and follow up	NA
Patrick O. Myers	2010	Europe	78	12 ± 3.5	OS	Preoperation and follow up	Preoperation, discharge and follow up

*OS* overall survival, *AIG* aortic insufficiency grade, *AVG* aortic valve gradient, *NA* not available.

### Pooled rate of mortality and survival

**Figure 2:**
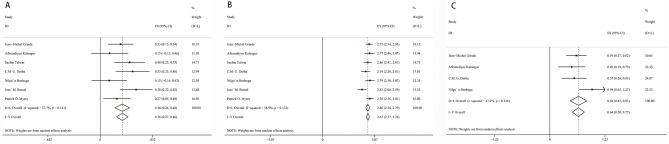
Forest plot of pooled hospital mortality (**A**), 5-year survival (**B**) and 5-year reoperation rates (**C**). *ES* effect size, *CI* confidence interval.

Arcsine conversion was used to pool the rate of mortality and survival. The pooled effect size (ES) of hospital mortality (Fig. 2A), 5-year survival (Fig. 2B) and 5-year reoperation (Fig. 2C) were 0.36 (95% CI 0.27–0.46, I^2^ = 37.7%, *p* < 0.001), 2.67 (95% CI 2.57–2.76, I^2^ = 38.9%, *p* < 0.001) and 0.64 (95% CI 0.5–0.77, I^2^ = 47.9%, *p* < 0.001), respectively. After back conversion, the eventual result of the pooled hospital mortality, 5-year survival and 5-year reoperation were 3.2% (95% CI 1.8–5.2%), 94.5% (95% CI 92.1–96.4%) and 9.9% (95% CI 6.1–14.1%), respectively.

### AIG and AVG between pre- and post-operation

**Figure 3:**
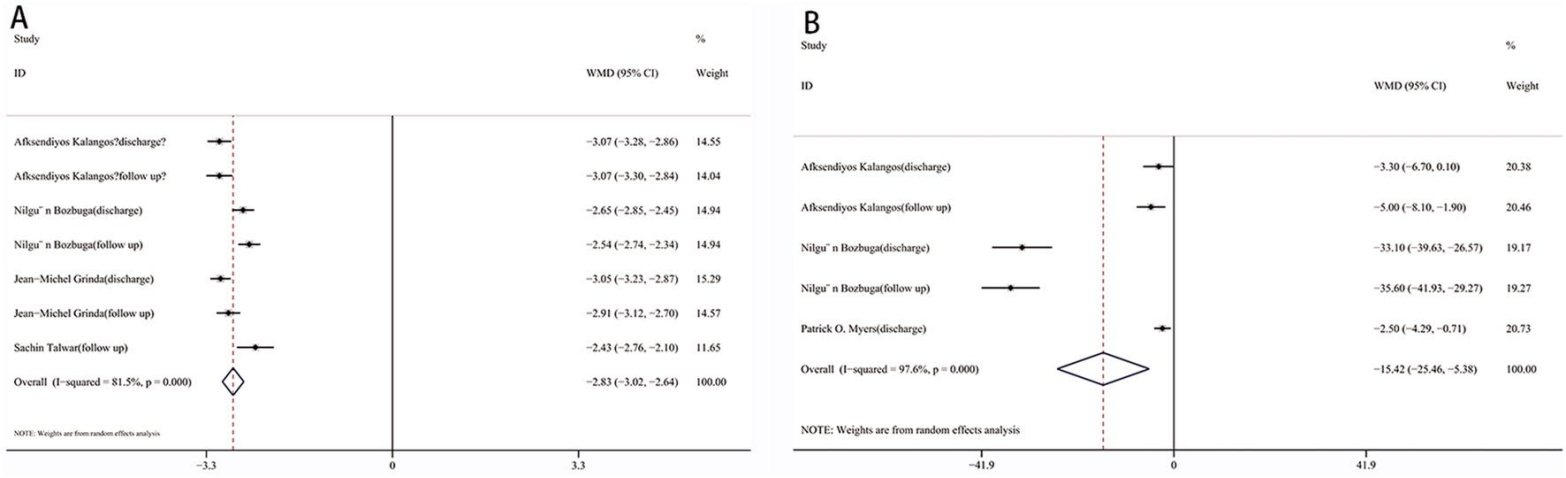
Forest plot of WMD of AIG (**A**) and AVG (**B**) between pre- and post-operation. *WMD* weighted mean differences, *CI* confidence interval.

**Figure 4:**
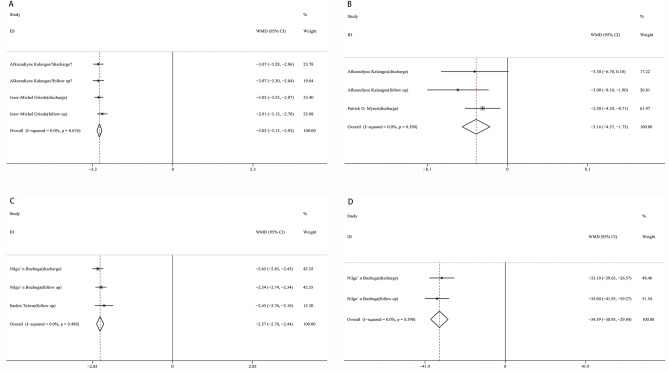
Subgroup analysis of AIG and AVG between pre- and post-operation: (**A**) AIG in European group; (**B**) AVG in European; (**C**) AIG in Asian group; (**D**) AVG in Asian group. *WMD* weighted mean differences, *CI* confidence interval.

Overall, the AIG and AVG were improved by 2.83 grades (95% CI 2.64–3.02, I^2^ = 81.5%, *p* < 0.001, Fig. 3A) and 15.42 mmHg (95% CI 5.38–25.46, I^2^ = 97.6%, *p* = 0.003, Fig. 3B) after the operation. After assessing the heterogeneity, the studies were divided into two groups based on continent. In the European group, the AIG decreased by 3.03 grades (95% CI 2.92–3.13, I^2^ = 0%, *p* < 0.001, Fig. 4A) and the AVG decreased by 3.16 mmHg (95% CI 1.75–4.57, I^2^ = 0%, *p* < 0.001, Fig. 4B) after the operation. In the Asian group, the AIG and AVG were improved by 2.57 grades (95% CI 2.44–2.70, I^2^ = 0%, *p* < 0.001, Fig. 4C) and 34.39 mmHg (95% CI 29.84–38.93, I^2^ = 0%, *p* < 0.001, Fig. 4D), respectively.

### AIG and AVG between discharge and follow up

**Figure 5:**
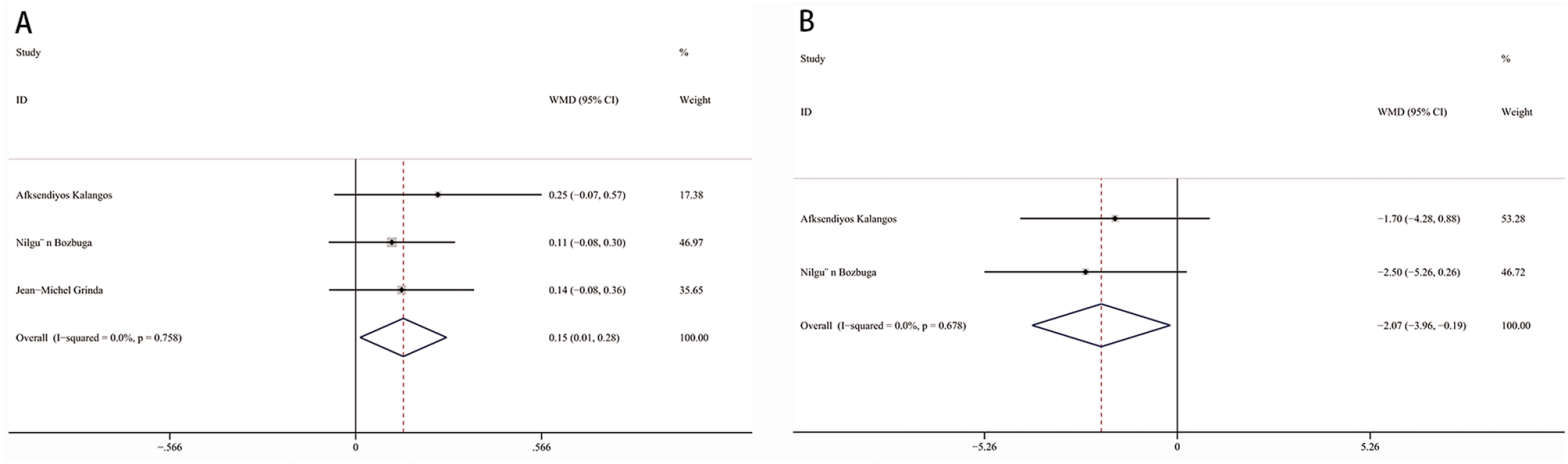
Forest plot of WMD of AIG (**A**) and AVG (**B**) between discharge and follow up. *WMD* weighted mean differences, *CI* confidence interval.

AIG and AVG were also analysed between discharge and follow up to assess the duration of the operation in improving aortic insufficiency and stenosis. Compared with discharge, the AIG increased by 0.15 grades (95% CI 0.01–0.28, I^2^ = 0%, *p* = 0.031, Fig. 5A) while the AVG was decreased by 2.07 mmHg (95% CI 0.19–3.96, I^2^ = 0%, *p* = 0.031, Fig. 5B) at the time of follow up.

### Quality assessment

The results of quality assessment of each study were shown in Table 2. The quality of each stud were divided into three groups by their scores: ≥ 7: good, 4–7: medium, < 4: poor.

**Table 2:** Quality assessment of each study.

First author	Article types	Comparison	Data integrity	Follow-up completed	Follow-up time	Quality
Jean-Michel Grinda	1	1	1	1	1	Medium
Afksendiyos Kalangos	1	1	2	1	2	Good
Sachin Talwar	1	1	1	1	3	Good
C.M. G. Durba	1	0	1	1	2	Medium
Nilgün Bozbuga	1	1	2	1	2	Good
José M. Bernal	1	1	1	1	2	Medium
Patrick O. Myers	1	1	2	1	2	Good

### Sensitivity analysis and publication bias

**Figure 6:**
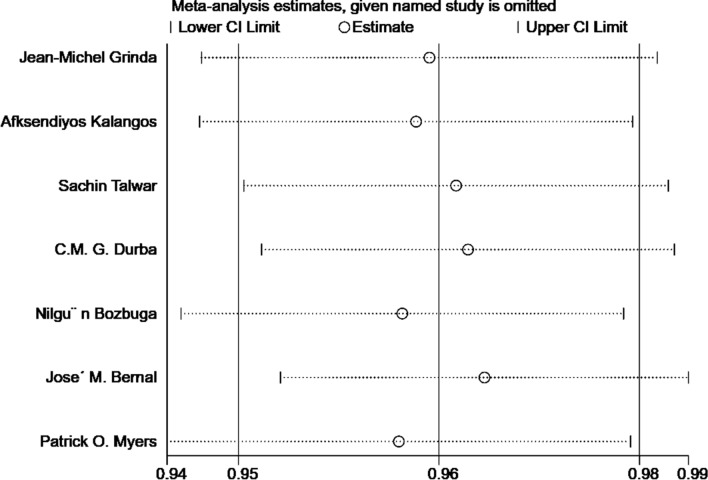
Sensitivity analysis of studies on hospital mortality.

Leave-one-out sensitivity analysis was conducted to evaluate the robustness of the results. Figure 6 shows that the hospital mortality could not be significantly changed by deleting any study, which indicated the robustness of the hospital mortality. Since less than 7 studies were included, the publication bias analysis does not lead to reliable results. Thus, the publication bias analysis was not conducted.

## Discussion

In the past decades, most rheumatic heart disease has disappeared in developed countries^15^, but it remains the main reason for cardiac surgery among poor countries and vulnerable populations in wealthy ones^16^. Valve replacement is the first choice during most of the operations. However, an increasing number of studies has indicated the feasibility of mitral valve repair in the rheumatic population^17,18^ in recent years, encouraging the attempts for rheumatic aortic valvuloplasty. Until now, studies focused on aortic valvuloplasty have not been systemically analysed. Therefore, we reviewed and summarized these studies for more accurate conclusions.

We used pooled hospital mortality, 5-year survival and 5-year reoperation to evaluate the safety and feasibility of aortic valvuloplasty surgery. The low hospital mortality and high 5-year survival reflected that valvuloplasty is safe and acceptable to improve rheumatic aortic insufficiency and stenosis. However, the high rate of reoperation within 5 years may prevent surgeons and patients from choosing this surgery. No patient would want to undergo another cardiac surgery within such a short time, and the second surgery is likely much more dangerous than the first. The main reasons for reoperation are as follows: (1) primary failure of aortic repair, (2) bacterial endocarditis, (3) valvuloplasty deterioration, (4) mitral dysfunction, and (5) tricuspid valve dysfunction. Aortic valve-related events are not the only reasons for reoperation.

AIG and AVG were used to evaluate the degree of aortic insufficiency and stenosis. Decreased AIG and AVG indicated that aortic insufficiency and stenosis were significantly improved after surgery. In subgroup analysis, the decrease in AIG in the European group was higher than that in the Asian group (3.03 grades in Europe and 2.57 grades in Asia). Conversely, the decrease in AVG was much higher in the Asia group. We carefully inspected the data and found that the mean AVG before the operation was much higher in Bozbuga’s study (mean AVG = 48.7 ± 21.2 mmHg) than that in the European group (12.5 ± 8.8 mmHg in Kalangos’s study and 18.1 ± 5.7 mmHg in Myers’s study). This may because Asia is less developed than Europe and patients in Asia often do not go to the hospital until they are seriously ill.

Comparing AIG and AVG between the time of discharge and follow up aimed to assess the duration of improving insufficiency and stenosis of the surgery. Compared with discharge, the AIG of follow up increased by 0.15 grades (95% CI 0.01–0.28, *p* = 0.031) and the AVG decreased by 2.07 mmHg (95% CI 0.19–3.96, *p* = 0.031) at the time of follow up. We found that the aortic insufficiency deteriorated but stenosis improved at the time of follow up compared with discharge, indicating that aortic valvuloplasty surgery has a more durable effect in improving stenosis than insufficiency.

Although there were no studies focused on the comparison between aortic valvuloplasty and other interventions, such as aortic valve replacement. A meta-analysis conducted by Wang^19^ indicated that mitral valve repair provides better short-term and long-term event-free survival for rheumatic patients. Which may provide the direction for the aortic valvuloplasty research.

This systematic review and meta-analysis had several limitations. First, the number of studies focused on rheumatic aortic repair was small, and only 7 studies and 418 patients were included in the study. Additionally, only 3 studies tested for both AIG and AVG before and after surgery among the 7 studies. Therefore, we had to integrate the AIG and AVG of discharge and follow up as postoperative data. Thus, one study was used twice (data of discharge and follow up) when comparing the AIG and AVG of pre- and post-operation. Second, all the included studies were retrospective observational studies and the conclusions of aortic valvuloplasty surgery did not have sufficient comparability. Third, the follow-up time of the studies was not long enough. Thus, the 5-year survival could not credibly describe the safety and effectiveness of aortic valvuloplasty surgery because cardiac patients had a much longer postoperative survival time than those who have cancer. Fourth, all the studies were from Asia and Europe; the conclusions might not apply to other populations.

## Conclusion

Aortic valvuloplasty surgery in patients’ rheumatic disease is safe and effective to improve rheumatic aortic insufficiency and stenosis, and it has a more durable effect in improving stenosis than in improving insufficiency. However, efforts should also be made to reduce the rate of reoperation. Considering the limitations of this study, more large-scale studies on various continents and ethnic groups are needed for more accurate conclusions of the value of aortic valvuloplasty surgery, as well as comparisons between aortic valve replacement and repair.

## Abbreviations

CI: Confidence interval
OS: Overall survival
AIG: Aortic insufficiency grade
AVG: Aortic valve gradient
WMD: Weighted mean differences
ES: Effect size
